# NON-SMALL-CELL LUNG CANCER: Real-World Population-Based Cohorts’ Study

**DOI:** 10.3390/cancers17040648

**Published:** 2025-02-14

**Authors:** Alessandra Buja, Massimo Rugge, Alberto Bortolami, Manuel Zorzi, Federico Rea, Anna Zanovello, Giovanna Scroccaro, Pierfranco Conte, Giulia Pasello, Valentina Guarneri, on behalf of Rete Oncologica Veneta, Periplo Foundation

**Affiliations:** 1 Department of Cardiologic, Vascular and Thoracic Sciences, and Public Health, University of Padova, Via Loredan, 18, 35100 Padova, Italy; federico.rea@unipd.it (F.R.); annazanovello2@gmail.com (A.Z.); 2 Department of Medicine DIMED—Pathology Unit University of Padova, 35100 Padova, Italy; massimo.rugge@unipd.it; 3 Coordinamento Regionale per le Attività Oncologiche (CRAO), Regione Veneto, 30100 Venezia, Italy; alberto.bortolami@regione.emilia-romagna.it (A.B.); giovanna.scroccaro@regione.veneto.it (G.S.); 4 Veneto Tumor Registry (RTV), Azienda Zero, 35100 Padova, Italy; manuel.zorzi@azero.veneto.it; 5 Camillo Hospital IRCCS, Regione Veneto, 30100 Venezia, Italy; 6 Periplo Foundation, 26100 Cremona, Italy; 7 Oncologia Medica 2, Istituto Oncologico Veneto, I.R.C.C.S., 35100 Padova, Italyvalentina.guarneri@unipd.it (V.G.); 8 Department of Surgery, Oncology and Gastroenterology, University of Padova, 35100 Padova, Italy

**Keywords:** lung cancer, NSCLC, real-world oncology, health care costs, population-based studies, health economics

## Abstract

Introduction: This retrospective follow-up study evaluates patient outcomes and treatment costs in two cohorts of incident NSCLC patients recorded in the population-based cancer registry of the Veneto Region in 2017 and 2019. Methods: This study examines two cohorts of incident NSCLC patients recorded by the Regional Veneto cancer registry in 2017 (493 patients) and 2019 (557 patients). Cancer patients were followed for three years after diagnosis to assess the disease’s outcomes and analyze the overall treatment-related costs. Overall survival and cancer-specific mortality were evaluated using Cox regression models. The log-transformed overall treatment costs for the 3 years following diagnosis were analyzed using linear regression. Results: The overall mortality risk significantly lowered in the 2019 cohort (HR 0.84; 95% CI 0.72–0.98, *p* = 0.024). Stage III patients were not associated with a significant overall survival rate (HR 0.71; 95% CI 0.50–1.02; *p* = 0.065) but were associated with significantly higher cancer-specific survival (HR 0.61; 95% CI 0.41–0.91; *p* = 0.015). Overall, the 2019 cohort showed significantly higher costs (coefficient 0.16; 95% CI 0.02–0.30, *p* = 0.025), particularly as a result of increases in the costs of drug administration, outpatient services, and medical devices. However, during the same 3-year follow-up period, this cohort featured lower average costs for hospice care and hospitalizations. Conclusions: These results revealed notable differences in clinical outcomes and patient-related costs in incident NSCLCs in 2017 and 2019. The study highlights the importance of monitoring clinical outcomes and management costs in real-world oncology practice.

## Simple Summary

This retrospective, 3-year follow-up study analyzed clinical outcomes and overall treatment costs in two cohorts of NSCLC patients recruited in 2017 and 2019. In real-life oncology practice, the study linked the 2019 cancer cohort to a significant improvement in cancer-specific survival, accompanied by only a slight increase in patient management costs. It was observed an increase in the costs of drug administration, outpatient services, medical devices and conversely in a decrease in hospital and hospice care costs. These results are likely related to changes in the care protocols implemented during that period.

## 1. Introduction

Lung cancer is a global health challenge, with an estimated 2 million new cases and over 1.5 million deaths globally each year [1,2,3]. Effective cancer prevention and treatment strategies require comprehensive approaches combining policies promoting accessibility with high-quality care [4].

Lung cancer prevention and treatment strategies are affected by their economic costs and operational plans. Targeted therapies have greatly enhanced the treatment options for non-small-cell lung cancer (NSCLC). However, rising cancer incidence, high costs of treatments, and the significant disabilities linked to NSCLC pose a substantial financial burden on public health initiatives [5,6]. These rising costs affect taxation and influence healthcare providers’ decisions.

Estimating cancer outcomes and treatment costs in the real world is crucial for shaping effectively oncology care and efficiently allocating public health economic resources.

This study aims to analyze clinical outcomes and treatment costs for two cohorts of newly diagnosed NSCLC patients recorded in the population-based cancer registry (RTV) of northeastern Veneto, Italy, in 2017 and 2019.

## 2. Methods

### 2.1. Socio-Economic and Healthcare Setting

The Italian National Health System is funded through general taxation and managed at the regional level. It operates based on ethical principles of universal coverage, fairness, unrestricted access, freedom of choice, and a diversity of service provision. Healthcare management is overseen by regional authorities in line with national reference criteria, ensuring equitable care provision across the country.

Health professionals (general practitioners, health professionals, etc.) of the regional territory (about 4,900,000 residents) provide primary care to their referring community. Secondary care is the responsibility of comprehensive community hospitals. Highly specialized institutions (e.g., cancer centers, teaching hospitals, officially recognized private providers) deliver tertiary health care interventions.

In 2013, the Regional Veneto Government established an interdisciplinary Oncology Network (ROV) committed to providing, implementing, and monitoring the diagnostic and therapeutic pathways to be applied in oncology patients. Based on the best national and international clinical evidence, dedicated documents outline criteria for cancer prevention, diagnostic procedures, treatment strategies, and end-of-life care for the most common types of cancer [7,8,9].

In 2022, the ROV released a detailed document addressing the PDTA dedicated to lung cancer patients, spanning from initial diagnosis to end-of-life support [10].

### 2.2. Study Population

This retrospective population-based cohort study analyzed 1077 incidents of non-small-cell lung cancer (NSCLC) diagnosed in 2017 (504 cases) and 2019 (573 cases) across two districts in the Veneto region of northeastern Italy. In both districts, cancer recording involved the entire resident population. The NSCLC clinical-pathological data were obtained from the high-resolution Veneto Tumors Registry, which included the clinical information collected within six months following the initial cancer diagnosis. Cases lacking sufficient information for correct staging were identified as undiagnosed and labeled as “missing stage”.

### 2.3. Costs Analysis

The cost analysis was conducted using anonymized aggregate data. For both patient cohorts, the cost estimates encompass a 3-year period following the initial cancer diagnosis. These estimates account for all disease-related expenses as provided by the Regional Health Authority. Table 1 outlines the sources and profiles of the aforementioned administrative data. Each patient was assigned a unique and anonymous identification code, which was used to link all administrative data covering hospital admissions, outpatient visits, drug prescriptions, emergency room visits, medical devices, hospice admissions, and vital statuses. The total costs were evaluated over 3 years following the diagnosis of NSCLC. The average per-patient costs were calculated and stratified according to the stage of disease at the time of diagnosis.

### 2.4. Statistical Analysis

Descriptive analyses were used to analyze the cost and survival characteristics of the sample. Kaplan–Meier curves were created to compare survival patterns at different stages by year. The log-rank test was used to compare the survival functions of patients diagnosed in 2017 and 2019 in each stage. A linear regression analysis was performed to test whether total costs (3 years after diagnosis of NSCLC), log-transformed, were associated with the index year, adjusting for year of diagnosis, sex, age, and stage at diagnosis. Cox regression models were run to study overall and lung cancer-specific mortality, adjusting for the year of diagnosis, sex, age, and stage at diagnosis. A further Cox regression analysis was conducted, stratified by stage and adjusted for year of diagnosis, sex, and age. The statistical packages R 3.6.2 and SAS 9.4 were used for the record linkage and all statistical analyses.

### 2.5. Ethics

The study was conducted following the principles established in the Declaration of Helsinki. All data were anonymized following Italian regulations and were handled for monitoring and quality assurance purposes. The data analyses were performed on anonymous aggregated data with no chance of individuals being identifiable. Ethical approval for the study was obtained from the Veneto Oncological Institute’s ethics committee (no. 03/2021).

## 3. Results

This population-based study included 1077 incidents of NSCLC diagnosed in residents of the Veneto Region in the years 2017 and 2019. A total of 504 cancer cases (47.0%) were diagnosed in 2017 and 573 (53.0%) in 2019. The cancer population included 669 males and 409 females (M:F = 1.63). The average age of all patients was 73.8 years (SD ± 11.1), with no difference between patients enrolled in 2017 and 2019 (year 2017 = 73.6; year 2019 = 74) (Table 2).

At enrollment, the patients’ distribution by clinicopathological TNM stage was as follows: stage I = 119 (11.0%); stage II = 60 (5.6%); stage III = 164 (15.2%); stage IV = 700 (62%). In 34 (3.2%) cancer patients, the tumor stage at initial diagnosis was unknown.

In the two considered patient cohorts, Table 3 shows the mean overall costs and the mean costs by cost item per patient. The reported costs refer to 3 years after cancer incidence. The table also reports the cost differences (delta values) by cohort (2017 versus 2019).

In the 2017 cohort, the average overall cost per patient over a 3-year follow-up period was EUR 45,590. In the 2019 cohort, this amount increased by EUR 2256, bringing the average cost to EUR 47,846. Specifically, the 2019 cohort experienced an increase in mean cost per patient across the following areas of medical interventions: (a) Medications rose by EUR 1547 (a 7.8% increase); (b) Outpatient services increased by EUR 1643 (an 18.0% increase); and (c) Medical devices saw a rise of EUR 250 (a 30% increase). Conversely, there was a downward trend in costs related to hospitalization (EUR 501; 4% reduction) and hospice-related costs (EUR 618; 51% reduction).

Table 4 summarizes the results of the regression analysis of 3-year costs per patient, adjusted for year of incidence, age, sex, and cancer stage at diagnosis. A statistically significant increase in mean costs was observed for the 2019 cohort (coeff. 0.16 IC 95% 0.02–0.30 *p* = 0.025). Moreover, the analysis revealed that costs decrease with age at diagnosis of patients (*p* < 0.001), and overall costs over a 3-year follow-up period are greater in patients in stage III at diagnosis compared to patients in stage I, but lower in patients in stage IV at diagnosis compared to those in stage I. In 2019, nearly 10% of stage III patients who did not undergo surgical treatment were treated with Durvalumab outside of clinical trials. The administration of targeted therapies did not differ in the two considered cohorts (2017: 43 patients [8.5%]; 2019: 48 patients [8.4%]). Immunotherapy prevailed significantly in the 2019 cohort (108 patients [18.8%]) versus 55 [10.9%], respectively).

In the 2017 cohort, the 3-year overall mortality rate was 77.6% (95% CI: 73.7–81.0); stage I: 22.8% [11.1–33.0], stage II: 24.0% [5.3–39.0], stage III: 74.4% [63.3–82.2], and stage IV: 92.2% [88.6–94.7]) In the 2019 cohort, the 3-year mortality rate was 77.0% (95% CI: 73.3–80.2); stage I: 13.3% [4.3–21.5], stage II: 44.1% [24.7–58.5], stage III: 64.1% [51.7–73.3], and stage IV: 93.0% [89.8–95.1]).

**Figure 1:**
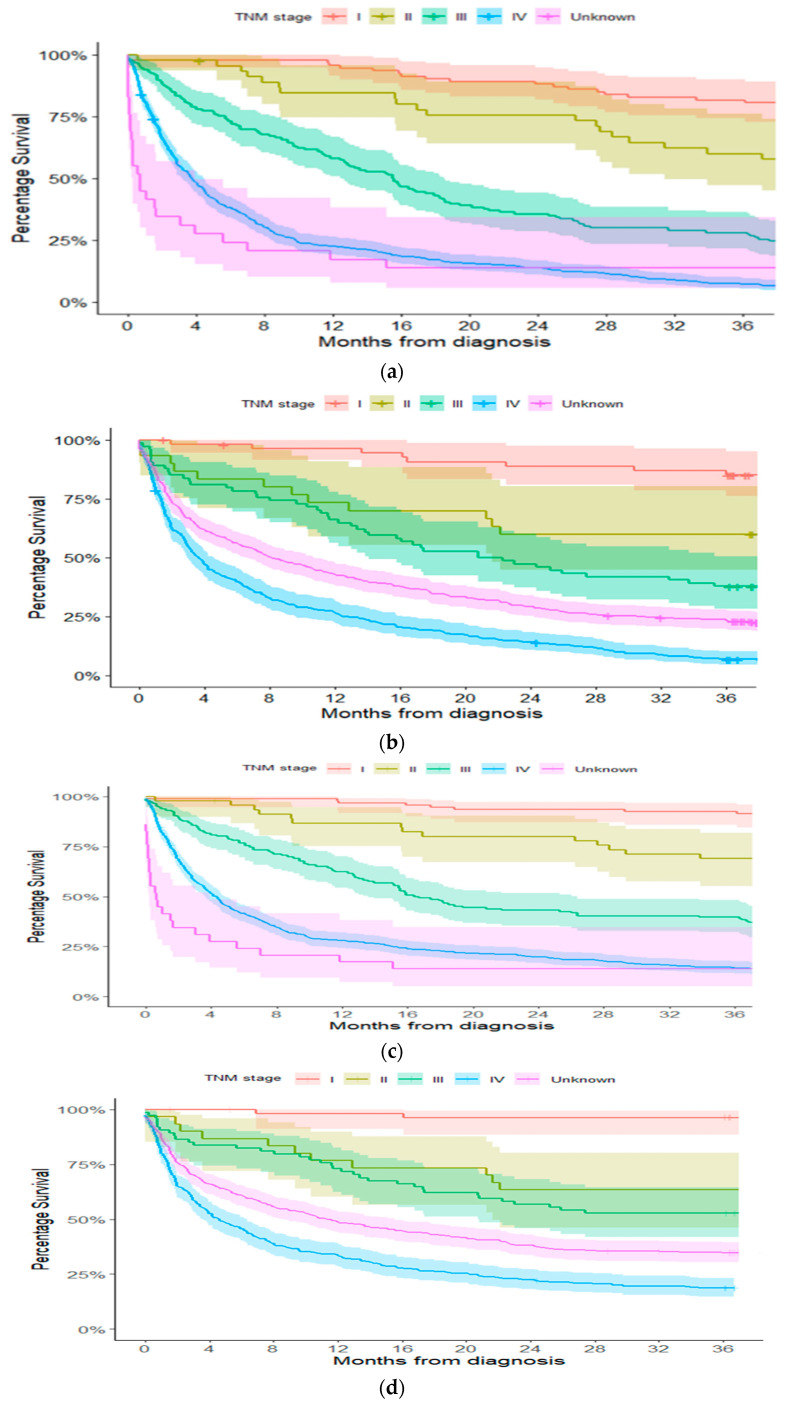

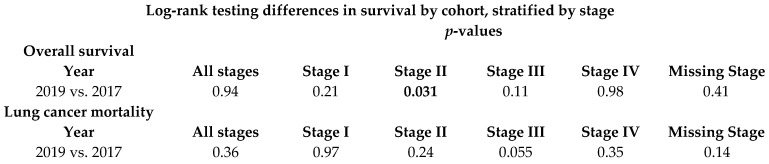
(**a**) Kaplan–Meier curve of overall survival up to 36 months from diagnosis, stratified by stage, for patients in the 2017 cohort. (**b**) Kaplan–Meier curve of overall survival up to 36 months from diagnosis, stratified by stage, for patients in the 2019 cohort. (**c**) Kaplan–Meier curve of lung cancer-specific mortality up to 36 months from diagnosis, stratified by stage, for patients in the 2017 cohort. (**d**) Kaplan–Meier curve of lung cancer-specific mortality up to 36 months from diagnosis, stratified by stage, for patients in the 2019 cohort.

Figure 1a–d show the Kaplan–Meier survival curves for overall and NSCLC-specific mortality stratified by stages in the 2017 and 2019 cohorts. The log-rank test indicated a significant improvement in overall survival for stage II patients (*p* = 0.031) and a not-significant improvement in NSCLC-specific survival for stage III patients (*p* = 0.055).

Table 5 presents the results of the Cox regression analysis for overall and disease-specific mortality. The 2019 cohort showed a significantly lower mortality hazard (HR 0.84, 95% CI 0.72–0.98, *p* = 0.024).

Cox regression analyses stratified by cancer stage (adjusted for sex and age at diagnosis) revealed that the overall survival for stage III approached statistical significance (HR 0.71, 95% CI 0.50–1.02, *p*= 0.065). Furthermore, a statistically significant improvement in lung cancer-specific survival for stage III was observed (HR 0.61, 95% CI 0.41–0.91, *p*= 0.015). Other stages showed no significant differences in survival rates.

## 4. Discussion

This retrospective, 3-year follow-up study analyzed clinical outcomes and overall treatment costs in two cohorts of NSCLC patients recruited in 2017 and 2019. In real-life oncology practice, the study linked the 2019 cancer cohort to a significant improvement in cancer-specific survival, accompanied by only a slight increase in patient management costs. These results are likely related to changes in the care protocols implemented during that period.

The cancer-specific survival improvement plausibly results from the combined benefits of the earlier Durvalumab implementation in stage III disease, and, even more, the broader administration of immunotherapy to patients progressing to stage IV. Durvalumab has proven to be an important and beneficial option as standard maintenance therapy for stage III disease. Clinical trials and real-world studies conducted since 2019 show that approximately 10% of patients with untreated stage III NSCLCs have received Durvalumab with a significant advantage in preventing disease progression [11,12]. According to the present findings, the rate of immunotherapy nearly doubled from 10.9% to 18.8% between 2017 and 2019. This trend reflects the expanded implementation of immune checkpoint inhibitors, resulting in extended survival and improved quality of life of advanced NSCLC patients [13,14].

Over the 3 years following diagnosis, the 2019 cohort showed a slight increase in the average total cost per patient, amounting to less than EUR 2500. Comparing costs between 2017 and 2019, the study documented that expenses for inpatient medications and outpatient services rose while hospitalization and hospice care costs declined. These trends in NSCLC management reflect a broader shift in oncology care, primarily due to the adoption of innovative therapies, particularly immunotherapy. This rearrangement has improved survival rates, enhanced quality of life, and reduced resource utilization, especially in end-of-life care [15,16].

A critical analysis of cost allocation changes offers valuable insights. The trend towards acute hospitalization indicates a shift in approach to oncology healthcare, moving from inpatient treatments to outpatient management of cancer and its symptoms [17]. The reduction in hospitalization and hospice costs, in particular, makes plausible an improvement in patients’ quality of life by extending cancer care interventions beyond mere survival rates to encompass overall patient well-being [18].

Waterhouse et al. noted that the costs associated with immunotherapy are significantly higher than those of conventional chemotherapy. Although lower hospitalization rates help to partially offset this increase, the overall economic burden remains greater. A similar study conducted in Catalonia revised data on patients diagnosed with NSCLC from the tumor registry of the University Hospital of Vic from 2002 to 2021 and evaluated survival outcomes and data on pharmacological costs, finding an increase in survival 18 months after diagnosis in patients with advanced stages, along with the advent of immunotherapy and an increase in drug costs, which reached a peak of EUR 48,283.80 in 2017 after the advent of immunotherapy [19]. Nevertheless, studies indicate that immunotherapies may be cost-effective in the long run [20]. Wan et al. found that nivolumab for previously treated advanced NSCLC is cost-effective compared to docetaxel, with an incremental cost-effectiveness ratio of USD 57,933 per QALY gained [21]. However, the cost-effectiveness often depends on factors such as PD-L1 expression levels and the specific immunotherapy agent used [22]. Regarding the utilization of Durvalumab as maintenance therapy for unresectable stage III NSCLC, a recent paper found that the therapy was found to be cost-prohibitive from the perspective of various international payers according to country-specific willingness-to-pay thresholds per QALY [23]. Another real-word research study demonstrated a superior cost-effectiveness of Atezolizumab compared to that of Durvalumab [24]. The rising costs, however, can be attributed not only to the advent of novel targeted therapies, but also, accounting for half of the total increment, to the corresponding increase in the utilization of diagnostic tests to identify suitable candidates for these treatments. A major contributor to the escalating expenses is the expansion of molecular marker testing. There was a notable increase in the use of other molecular profiling methods to guide treatment selection. A study [25] highlighted the growing adoption of molecular marker testing, which has led to increased costs but also improved patient outcomes through personalized treatment approaches. The rising costs associated with healthcare devices can be partly attributed to the decreasing demand for hospice care services, with a reduction of costs for these structures [26] and, consequently, there has been a corresponding increase in the demand for specialized devices and equipment delivered at home [27]. One of the primary drivers of this cost increase is the need for hospital-grade beds and mattresses in home settings, as traditional home beds may not provide the necessary support and pressure relief [28]. Another significant cost factor is the need for patient transfer and mobility devices in home settings [29]. Furthermore, the use of incontinence products like diapers has also contributed to the increased costs associated with home-based care. It is important to note that while these increased costs may pose a financial challenge for healthcare providers, the investment in specialized devices and equipment is often justified by the improved patient experience and quality of care provided in home-based care. Receiving care in the comfort of one’s home can have significant psychological and emotional benefits for patients and their families [30], and the appropriate use of specialized devices can contribute significantly to achieving these goals.

The current study offers valuable observational insights into oncology practice, which differs significantly from the “rigorous” experimental settings of controlled clinical trials [31,32]. Real-world findings complement the results of randomized controlled trials by profiling the long-term effectiveness and economic impact of new clinical pathways in broad cancer populations. Real-world data-based measures serve as an invaluable resource, informing clinicians, patients, and healthcare policymakers about the performance of clinical interventions outside of controlled research settings. Furthermore, real-world evidence is crucial for health technology assessments conducted by national and regional institutions, which guide clinical decision-making [33].

One limitation of this study is the possibility of overlooking significant indirect costs. Additionally, the study did not account for experimental therapies that are provided free of charge, leading to these costs not being recorded in the regional administration database. Furthermore, as new oncological therapies are continuously introduced, this evolving landscape is expected to have a considerable impact on stage-specific survival rates and the overall economic implications of drug-related expenses. Moreover, the difference in survival between the two cohorts could be attributed to factors other than treatment management, such as heterogeneity in the distribution of different confounding factors, such as stage at diagnosis. Although our analyses were adjusted for the stage at diagnosis, other factors like differences—even within the same hospital context—in equipment, time process management, or patient comorbidities might have played a role. Comorbidities significantly impact survival outcomes, as they have significant implications for the prognosis and treatment of lung cancer patients. Future real-world studies should stratify patients based on these factors to provide more nuanced insights. Finally, since this study was conducted in a specific Italian region with one of the top regional healthcare systems, the results may not be generalizable to other regions or countries with different treatment protocols and cost reimbursement structures. Similar studies should be conducted in other regions or countries to validate our findings.

In conclusion, the current findings show that most recently diagnosed NSCLC patients have experienced improved survival rates and only slight increases in the costs of their clinical management. This study highlights the importance of evaluating both clinical outcomes and related costs at the population level in oncology. The significant impact on public health services and healthcare budgets is essential for real-world assessments of the cost-effectiveness of cancer treatments and their overall burden.

## Figures and Tables

**Table 1:** Healthcare costs of NCLSC patients; administrative regional databases included in the cost estimates.

Administrative Databases	Data Collection
Hospital admissions	Defines the Diagnosis-Related Groups (DRGs) for each admission, valued according to an inpatient formulary (i.e., Tariffario Prestazioni Ospedaliere), encompassing all in-hospital activities, including drugs.
Outpatient visits	Procedures/services provided under regional health service funding at outpatient facilities. Economic values based on rates established by an outpatient formulary (i.e., Tariffario Prestazioni Ambulatoriali).
Emergency room admissions	Costs are based on the rates for all medical procedures and interventions performed during A&E visits.
Pharmaceutical distribution and hospital drug consumption	Consider the costs of medical therapies (costs calculated on the prescribed doses).
Medical devices	Reports the expenditures incurred by regional authorities for the provision of medical devices.
Hospice admission	Costs are calculated by multiplying a regional daily rate by the number of days spent in hospice.

**Table 2:** NSCLCs indexed by the year of diagnosis.

		2017 Cohort: 573 Patients		2019 Cohort: 404 Patients		OVERALL: 1077 Patients	
AGE		Mean	SD	Mean	SD	Mean	SD
		73.6	10.9	74	10.9	73.8	11.1
SEX		n	%	n	%	n	%
	Females	190	37.7	218	38	408	37.9
	Males	314	62.3	355	62	669	62.1
CANCER STAGE	I	57	11.3	62	11	119	11
	II	26	5.2	34	6	60	5.6
	III	86	17.1	78	14	164	15.2
	IV	322	63.9	378	66	700	65
	Missing	13	2.6	21	4	34	3.2

**Table 3:** 3-year costs by patient according to the year of NSCLC incidence: 2017 cohort versus 2019 cohort.

	Year 2017	Year 2019	DELTA 2019 versus 2017
HOSPITALIZATION	11,469.2	10,968.7	−500.54
INPATIENT DRUG	20,718.6	22,266.6	1547.96
EMERGENCY ROOM VISITS	623	683.4	60.44
OUTPATIENT DRUGS	1581.8	1455.7	−126.08
OUTPATIENT SERVICES	9161	10,805	1643
MEDICAL DEVICES	814.5	1065	250.21
HOSPICE	1221.97	603.13	−618.84
TOTAL	45,590.46	47,846.74	2256.28

**Table 4:** Linear regression: dependent variable total costs (euros) 3 years from diagnosis (logarithmically transformed).

	Coefficient	C.I. 95%	SE	*p*-Value
YEAR (reference: year 2017)				
2019	0.16	0.02–0.30	2.25	0.025
AGE AT DIAGNOSIS	−0.03	−0.03–0.02	0.10	<0.001
SEX (reference: Female)				
Male	−0.10	−0.25–0.04	2.33	0.164
CANCER STAGE (reference: Stage I)				
Stage II	0.33	−0.03–0.69	5.80	0.068
Stage III	0.29	0.01–0.56	4.42	0.039
Stage IV	−0.27	−0.50–0.05	3.69	0.018
Stage missing	−0.26	−0.74–0.22	7.71	0.283

**Table 5:** Cox regression: overall and NSCLC-specific survival.

OVERALL SURVIVAL-	HR	C.I. 95%	SE	*p*-Value
YEAR (reference: year 2017)				
2019	0.92	0.80–1.06	0.07	0.235
AGE AT DIAGNOSIS	1.03	1.03–1.04	0.00	<0.001
SEX (reference: Female)				
Male	1.29	1.12–1.49	0.07	0.001
CANCER STAGE (reference: Stage I)				
Stage II	2.03	0.49–1.21	0.27	0.008
Stage III	4.94	0.20–3.29	0.21	<0.001
Stage IV	11.02	0.09–7.55	0.19	<0.001
NSCLC-SPECIFIC SURVIVAL-				
YEAR (reference: year 2017)				
2019	0.84	0.72–0.98	0.08	0.024
AGE AT DIAGNOSIS	1.02	1.01–1.03	0.00	<0.001
SEX (reference: Female)				
Male	1.20	1.03–1.41	0.08	0.023
CANCER STAGE (reference: Stage I)				
Stage II	3.53	1.65–7.56	0.39	0.001
Stage III	10.11	5.38–19.03	0.32	<0.001
Stage IV	21.32	11.55–39.39	0.31	<0.001

## Data Availability

The data supporting the findings of this study are held by the Veneto Epidemiological Registry and were used under license for the present work, but they are not publicly available. These data are nonetheless available from Manuel Zorzi on reasonable request and subject to permission to be obtained from the Veneto Epidemiological Registry (Veneto Regional Authority).
